# Case Report: Immune Dysregulation Due to *Toxoplasma gondii* Reactivation After Allogeneic Hematopoietic Cell Transplant

**DOI:** 10.3389/fped.2021.719679

**Published:** 2021-08-10

**Authors:** Robert B. Lindell, Michael S. Wolf, Alicia M. Alcamo, Michael A. Silverman, Daniel E. Dulek, William R. Otto, Timothy S. Olson, Carrie L. Kitko, Paisit Paueksakon, Kathleen Chiotos

**Affiliations:** ^1^Department of Anesthesia and Critical Care Medicine, Division of Critical Care Medicine, Children's Hospital of Philadelphia and the University of Pennsylvania Perelman School of Medicine, Philadelphia, PA, United States; ^2^Department of Pediatrics, Division of Pediatric Critical Care Medicine, Monroe Carell Jr. Children's Hospital at Vanderbilt and the Vanderbilt University School of Medicine, Nashville, TN, United States; ^3^Department of Pediatrics, Division of Infectious Diseases, Children's Hospital of Philadelphia and the University of Pennsylvania Perelman School of Medicine, Philadelphia, PA, United States; ^4^Department of Pediatrics, Division of Pediatric Infectious Diseases, Monroe Carell Jr. Children's Hospital at Vanderbilt and the Vanderbilt University School of Medicine, Nashville, TN, United States; ^5^Department of Pediatrics, Division of Oncology, Children's Hospital of Philadelphia and the University of Pennsylvania Perelman School of Medicine, Philadelphia, PA, United States; ^6^Department of Pediatrics, Division of Pediatric Hematology and Oncology, Monroe Carell Jr. Children's Hospital at Vanderbilt and the Vanderbilt University School of Medicine, Nashville, TN, United States; ^7^Department of Pathology, Microbiology, and Immunology, Monroe Carell Jr. Children's Hospital at Vanderbilt and the Vanderbilt University School of Medicine, Nashville, TN, United States

**Keywords:** hematopoietic cell transplant, toxoplasmosis, immune dysregulation, multiple organ dysfunction syndrome, hyperferritinemia

## Abstract

Disseminated toxoplasmosis is an uncommon but highly lethal cause of hyperferritinemic sepsis after hematopoietic cell transplantation (HCT). We report two cases of disseminated toxoplasmosis from two centers in critically ill adolescents after HCT: a 19-year-old who developed fever and altered mental status on day +19 after HCT and a 20-year-old who developed fever and diarrhea on day +52 after HCT. Both patients developed hyperferritinemia with multiple organ dysfunction syndrome and profound immune dysregulation, which progressed to death despite maximal medical therapies. Because disseminated toxoplasmosis is both treatable and challenging to diagnose, it is imperative that intensivists maintain a high index of suspicion for *Toxoplasma gondii* infection when managing immunocompromised children, particularly in those with known positive *T. gondii* serologies.

## Introduction

*Toxoplasma gondii* is an intracellular protozoan parasite transmitted by meat containing tissue cysts and through fecal–oral transmission that typically causes an asymptomatic or mild self-limited infection (1). While the population prevalence of toxoplasmosis is decreasing, substantial regional heterogeneity in seroprevalence remains (2, 3). Among immunocompromised patients, toxoplasmosis can cause a disseminated primary infection, but most often represents reactivation of a prior infection. Neurologic findings are the most common clinical manifestations (4).

Toxoplasmosis after hematopoietic cell transplantation (HCT) generally occurs within 6 months of transplant (5). Features of toxoplasmosis typically include fever and non-specific signs, including respiratory and neurologic symptoms that overlap with other opportunistic infections (6). Serologic testing for *T. gondii* can be unreliable in immunocompromised patients; thus, diagnosis is often made by polymerase chain reaction (PCR) testing or microscopic identification of parasites in blood or tissue (7, 8). Pre-transplant serologic testing can identify patients at risk of reactivation, which may prompt earlier diagnostic evaluation. Exposed patients may benefit from PCR-based surveillance for *T. gondii* (9).

Here, we report two cases of disseminated toxoplasmosis in critically ill adolescents after HCT presenting with hyperferritinemic sepsis and multiple organ dysfunction syndrome (MODS). We review the heterogeneity of their presentation, treatment, and outcome and highlight the importance of prompt diagnosis and treatment of this highly lethal condition. This report is considered not a research on human subjects by both Institutional Review Boards (IRBs); consent was not required by the IRB and not obtained from the decedents.

## Case 1

A 19-year-old male with relapsed Philadelphia chromosome-like B cell acute lymphoblastic leukemia who received a haploidentical stem cell transplant developed fever on post-transplant day +16. Blood culture was positive for *Bacillus cereus*, which was treated with vancomycin and cefepime.

**Figure 1:**
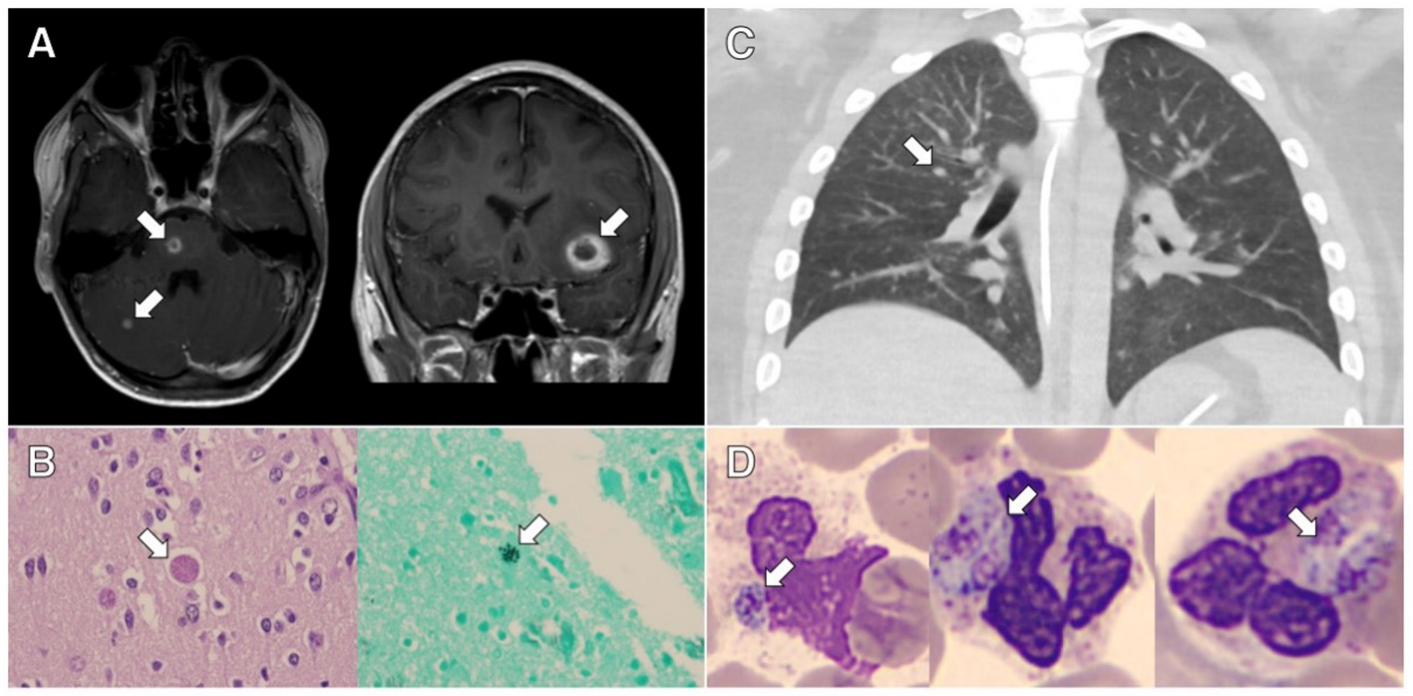
Clinical and pathological evidence of disseminated toxoplasmosis from the cases presented. **(A)** Axial and coronal contrast-enhanced magnetic resonance imaging of the brain from case 1 showing rim-enhancing lesions. **(B)** Representative slides stained with hematoxylin and eosin (HE; *left image*) and Grocott's methenamine silver stain (*right image*) showing cysts containing *Toxoplasma* bradyzoites in the brain tissue from case 1. **(C)** Axial contrast-enhanced chest computed tomography from case 2 showing bilateral centrilobular nodules and ground glass opacities. **(D)** Representative slides stained with HE showing neutrophils containing *Toxoplasma* tachyzoites in peripheral blood from case 2.

On day +17, he developed a headache and became somnolent. Computed tomography (CT) scan of the head showed a small left frontal intraparenchymal hemorrhage. Magnetic resonance imaging (MRI) of the brain revealed multiple ring-enhancing lesions consistent with possible septic emboli, shown in Figure 1A. Magnetic resonance angiography did not show evidence of intracranial aneurysm. Transthoracic echocardiogram showed no evidence of intracardiac shunt, valve disease, or vegetation. The patient was broadened to vancomycin and meropenem for treatment of presumed disseminated *B. cereus*. Due to persistent fever despite bloodstream clearance of *B. cereus*, MRI with ring-enhancing lesions, and the patient's known immunoglobulin G (IgG) seropositivity for *T. gondii*, trimethoprim/sulfamethoxazole (TMP/SMX) was started empirically from days +22 to +25. This was discontinued given the negative *T. gondii* PCR testing from blood, cerebrospinal fluid, and bronchoalveolar lavage (BAL) specimens. Diagnostic workup was negative for *Aspergillus, Pneumocystis jirovecii, Cryptococcus*, and viral infection.

Given the ongoing fever, development of vomiting, and diarrhea, an endoscopy was performed on day +49. Methylprednisolone was given from day +49 to +52 for empiric management of gastrointestinal graft-vs.-host disease (GVHD), but pathology showed rare apoptotic bodies in the duodenum and colon and did not support a diagnosis of GVHD.

On day +51, the patient developed rapid-onset respiratory failure and progressive somnolence. CT scan of the chest showed diffuse ground glass opacities. He was transferred to the pediatric intensive care unit (PICU), where he developed MODS with severe hypoxemic respiratory failure and septic shock requiring mechanical ventilation and significant vasoactive support. He was treated empirically with gentamicin, meropenem, TMP/SMX, amphotericin, cidofovir, albendazole, and ivermectin, with plan to start pyrimethamine/sulfadiazine when available. PCR testing of repeat BAL fluid identified *T. gondii*. A splenic lesion was also identified on ultrasound and biopsied, with PCR testing positive for *T. gondii*. All other bacterial, viral, and fungal studies were negative. TMP/SMX was dose-adjusted for the treatment of disseminated toxoplasmosis.

**Figure 2:**
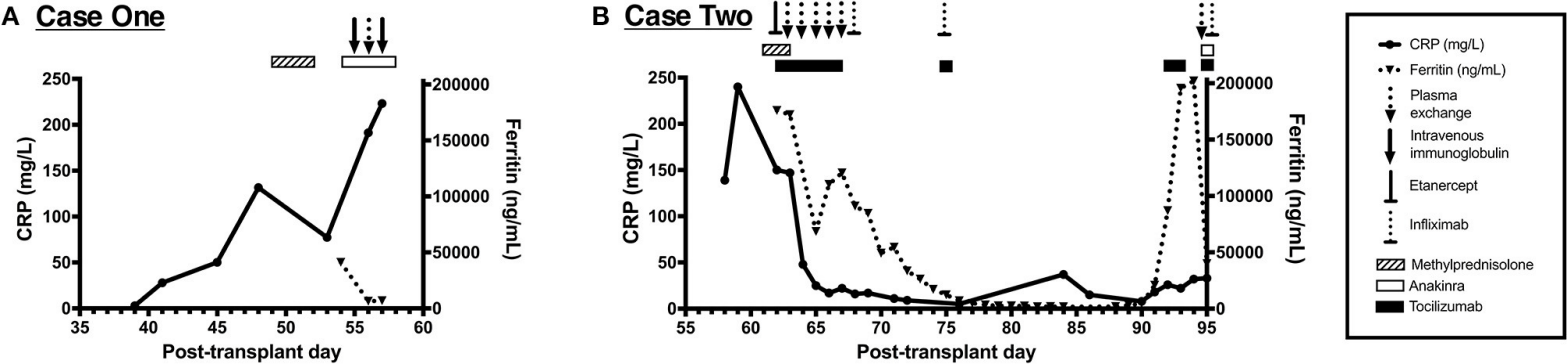
Immune dysregulation and exposure to associated immunomodulatory therapies from the cases presented. **(A)** Timeline of C-reactive protein (CRP) and ferritin values from case 1 showing progressive immune dysregulation. This patient was exposed to methylprednisolone, anakinra, intravenous immunoglobulin (IVIG), and plasma exchange, as shown *along the top of the panel*. **(B)** Timeline of CRP and ferritin values from case 2 showing two distinct periods of immune dysregulation. This patient was exposed to methylprednisolone, tocilizumab, anakinra, etanercept, infliximab, and plasma exchange, as shown *along the top of the panel*.

In addition to antimicrobials, the patient was concurrently treated with plasma exchange and intravenous immunoglobulin for thrombocytopenia-associated multiple organ failure from day +55 to +57. Despite therapy, he developed hyperferritinemia (peak level, 49,000 ng/mL) with concurrent hepatobiliary dysfunction and coagulopathy. The results of his clinical cytokine panel from day +54 are shown in Table 1. He was also treated with anakinra based on a reanalysis of a phase III randomized trial showing a mortality benefit of interleukin-1 receptor antagonist treatment in adult severe sepsis (10). A timeline of his immune dysregulation and associated therapeutics is shown in Figure 2A.

**Table 1:** Results from cytokine panels obtained clinically to guide immunomodulatory therapies.

**Cytokine (pg/mL)**	**Case 1:** **day +54**	**Case 2:** **day +64**	**Case 2:** **day +92**
IL-1β	<5	<5	<5
IL-2	<5	<5	<5
IL-4	75	<5	<5
IL-5	5	21	21
IL-6	4,353	45	170
IL-8	154	86	<5
IL-10	60	184	26
IL-12	–	<5	<5
IL-13	–	<5	<5
IL-18	8,111	–	–
IFN-γ	15	<5	13
TNF-α	2	–	–
GMCSF	2	–	–
CXCL9	1,681	–	–
sCD163	5,575	–	–
sIL-2R	5,835	9,330	2,739

*IL, interleukin; IFN, interferon; TNF, tumor necrosis factor; GMCSF, granulocyte–macrophage colony-stimulating factor; CXCL, C–X–C motif chemokine ligand; CD, cluster of differentiation.*

Given the patient's declining respiratory status and persistent MODS despite maximal support, his family elected to discontinue life-sustaining therapy on day +58. Autopsy findings were notable for evidence of disseminated toxoplasmosis, as shown in Figure 1B. Numerous inflammatory foci and *T. gondii* bradyzoites were seen in the myocardium and diaphragm. Neuropathologic evaluation revealed numerous hemorrhagic subcortical infarcts containing granulomatous inflammation and *T. gondii* bradyzoite cysts.

## Case 2

A 20-year-old male with immune-mediated aplastic anemia refractory to prior immune suppression therapy received an unrelated donor peripheral stem cell transplant. *Toxoplasma* serologies were not obtained during his pre-transplant evaluation as per institutional practice. His initial transplant course was uncomplicated, and he was discharged home on post-transplant day +21. On day +52, he was readmitted for fever and diarrhea. Due to fever and hypoxemia, a CT scan of the chest was obtained on day +55 and showed bilateral centrilobular nodules with right-sided predominance, as shown in Figure 1C. His infectious workup included multiple BALs, blood, and urine cultures and evaluation for *Pneumocystis jirovecii* and viral etiologies, which were all negative. He was maintained on broad-spectrum antibiotics and antifungal medications given the persistent fever despite negative cultures. Due to progressive hypoxemic respiratory failure, he was transferred to the PICU on day +61, where he required intubation and mechanical ventilation. Given the refractory hypoxemic respiratory failure, he was cannulated to venous–venous extracorporeal membrane oxygenation (ECMO) within 24 h of PICU transfer. His ECMO course was complicated by shock, hyperferritinemia, and acute kidney injury requiring renal replacement therapy.

Multimodal therapy for his immune dysregulation included pulse dose methylprednisolone (2 g/day) followed by a prolonged methylprednisolone course, plasma exchange, tocilizumab for IL-6 blockade, and etanercept for tumor necrosis factor alpha blockade, which was later transitioned to infliximab. A timeline of the immunomodulatory therapies is shown in Figure 2B. With improvement in his hyperinflammatory state, he was decannulated from ECMO on day +73.

On day +90, he had recurrence of fever without other changes in exam in the setting of a methylprednisolone taper; after a multidisciplinary discussion, tocilizumab, infliximab, and pulse dose methylprednisolone were administered for the management of immune dysregulation. The results of his clinical cytokine panel from day +64 to +92 are shown in Table 1. Despite these interventions, he had worsening hyperferritinemia by day +94 (peak level, 202,000 ng/mL), so anakinra was administered and plasma exchange was repeated. Despite immunomodulation, he continued to have refractory MODS with severe hypoxemic respiratory failure and catecholamine-refractory shock. At the request of his family, resuscitative efforts were discontinued on day +95. On the day of his death, *T. gondii* tachyzoites were noted on his peripheral blood smear (shown in Figure 1D), which were subsequently confirmed to be *T. gondii* by PCR and immunostaining. Postmortem analyses of the BAL specimens from day +60 and +65 were also positive for *T. gondii* by PCR. Postmortem *T. gondii* serologic testing was performed on a pre-transplant blood specimen and was positive for IgG.

## Discussion

Toxoplasmosis is a rare but severe complication after HCT that generally occurs within the first 6 months of transplant and is associated with substantial morbidity and mortality (3–5). These two cases highlight the potential for *T. gondii* reactivation to result in hyperferritinemic sepsis and MODS after HCT (11) and the lack of “classic” signs and symptoms of *T. gondii* in critically ill patients after HCT. Management of the immunocompromised patient with hyperferritinemic sepsis requires a highly collaborative approach, incorporating evaluation from critical care medicine, infectious disease, and stem cell transplant. Reaching a diagnosis is challenging but critical in these patients as some potential etiologies, including *T. gondii*, may be successfully treated if the diagnosis is made early in the clinical presentation. Several previous reports focused on pediatric HCT patients have characterized the association between toxoplasmosis and poor clinical outcomes, as summarized in Table 2.

**Table 2:** Previously reported cases of toxoplasmosis in adolescent and young adult patients (age ≤ 25 years) after HCT (since 2000).

**Authors/Reference**	**No. of patients**	**Age (years),** **sex**	**Date of onset** **(post-transplant)**	**Site of** **infection**	**Hospital** **survival**
De Medeiros et al. (12)	6	8, M	Not reported	CNS	Yes^a^
		11, M	Not reported	Disseminated	Yes^a^
		12, F	Not reported	Respiratory	No
		14, F	Not reported	CNS	No
		18, M	Not reported	CNS	No
		25, M	Not reported	CNS	Yes^a^
Duzovali et al. (13)	1	8, M	+76 days	CNS	Yes
Goebel et al. (14)	1	7, M	+16 days	Disseminated	No
Megged et al. (15)	1	12, F	+4 months	CNS	Yes^a^
Caselli et al. (16)	2	18, F	+16 months	CNS	Yes^a^
		20, F	+52 days	CNS	Yes
Kerl et al. (17)	1	15, F	+4 months	CNS	Yes
Rand et al. (18)	1	6, F	Not reported	Skin + Blood	Yes^a^
Yang et al. (19)	1	15, M	+66 days	Disseminated	No
Decembrino et al. (20)	3	6, M14, F16, M	+55 days+55 days+266 days	DisseminatedCNSCNS	NoYesNo
Czyzewski et al. (21)	3	9, M	+352 days	CNS + retina	Yes
		17, M	+90 days	CNS + retina	Yes
		17, F	+58 days	CNS	Yes
Year 2019; Komitopoulou et al. (22)	3	5, M	+40 days	Disseminated	No
		15, F	+2 months	CNS	Yes^a^
		16, F	+5 months	Disseminated	No
Sanchez-Petitto et al. (11)	1	25, F	+1 month	Disseminated	Yes

*M, male; F, female; CNS, central nervous system.*

a *Survived toxoplasmosis, but later died of another cause*.

In addition to progressive MODS, both patients developed significant immune dysregulation during their clinical course, as summarized in Figure 2. Immune dysregulation is a feature of certain forms of critical illness and is highly associated with mortality after sepsis (23), trauma (24), and cardiopulmonary bypass (25). Immunocompromised patients, including allogeneic HCT recipients, have an increased risk of immune dysregulation and mortality in the setting of critical illness (26, 27). In the case of toxoplasmosis, hyperferritinemia is thought to be precipitated by several *T. gondii* virulence factors that influence macrophage activation (28, 29). This activation also occurs in the microglia (30) and may explain the predominance of the neurologic signs and symptoms commonly seen in *T. gondii* reactivation.

Successful treatment of severe toxoplasmosis due to reactivation in immunocompromised patients is challenging, with limited data to support the recommended antibiotic regimens. The combination of pyrimethamine, sulfadiazine, and leucovorin is the recommended regimen for the treatment of toxoplasmosis reactivation in an immunocompromised host (31, 32). TMP/SMX is an alternative therapy if pyrimethamine is unavailable or not tolerated. Both regimens combine a sulfonamide antibiotic with a dihydrofolate reductase inhibitor. Two small, prospective randomized controlled trials comparing these combination agents have shown similar efficacy and a favorable side effect profile associated with the use of TMP/SMX (33, 34). Both pyrimethamine/sulfadiazine and TMP/SMX achieve adequate tissue exposure, including within the central nervous system (CNS) (35–37), and are treatments of choice for disease involving the CNS (32).

The diagnostic and management challenges inherent to critical illness after HCT also highlight the growing role for physicians with clinical expertise in pediatric onco-critical care. Immunocompromised children represent a small subset of patients with critical illness, but account for a substantial fraction of ICU mortality due to sepsis (27). As our mechanistic understanding of immune dysregulation in these high-risk patients continues to grow, we will have increasing opportunities to study the role of novel targeted therapeutics in modifying complex host–pathogen interactions. In the meantime, because toxoplasmosis is an uncommon but treatable cause of MODS, it is imperative that clinicians maintain a high index of suspicion for *T. gondii* infection when managing immunocompromised children with progressive organ failure despite maximal medical therapies, particularly those with serologic evidence of prior *T. gondii* exposure.

## Data Availability Statement

The original contributions generated for this study are included in the article/supplementary material, further inquiries can be directed to the corresponding author/s.

## Author Contributions

RL, MW, AA, MS, DD, WO, TO, CK, and KC provided patient care and contributed to the conception of this case report. PP prepared the neuropathology slides for this report. RL wrote the first draft. All authors contributed to manuscript revision, read, and approved the submitted version.

## Conflict of Interest

The authors declare that the research was conducted in the absence of any commercial or financial relationships that could be construed as a potential conflict of interest.

## Publisher's Note

All claims expressed in this article are solely those of the authors and do not necessarily represent those of their affiliated organizations, or those of the publisher, the editors and the reviewers. Any product that may be evaluated in this article, or claim that may be made by its manufacturer, is not guaranteed or endorsed by the publisher.
